# Supplementary figures and images for: Socioeconomic status and older adult’s experiences of weight loss: a qualitative secondary analysis
Source: PLoS One. 2025 Apr 22;20(4):e0321313. doi: 10.1371/journal.pone.0321313 (PMC12013945; doi:10.1371/journal.pone.0321313)

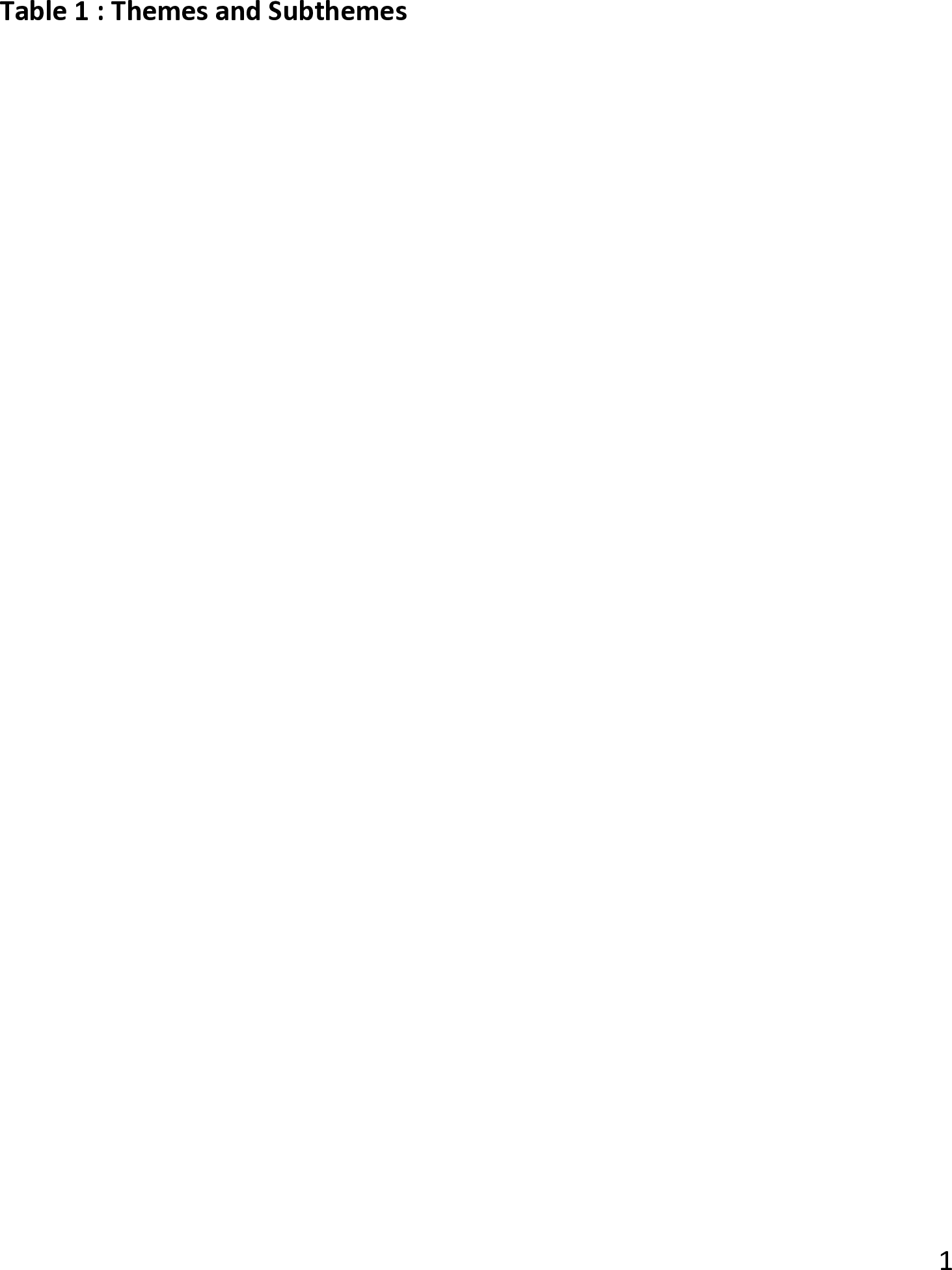

Supplement: S1 Table — (ZIP) [file pone.0321313.s001.zip › PACE Corrected/S1 table.tif]

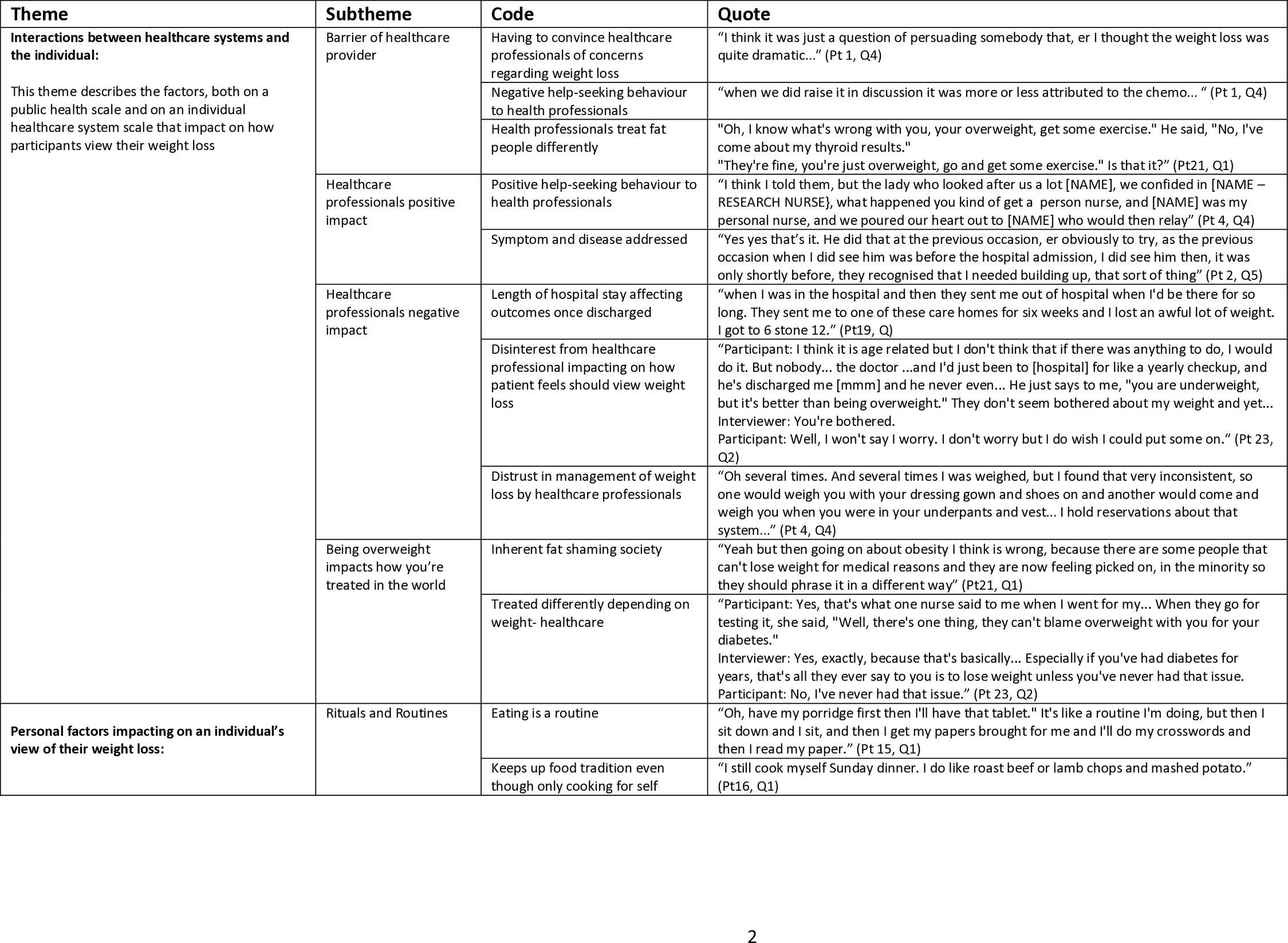

Supplement: S1 Table — (ZIP) [file pone.0321313.s001.zip › PACE Corrected/S1 table.tif]

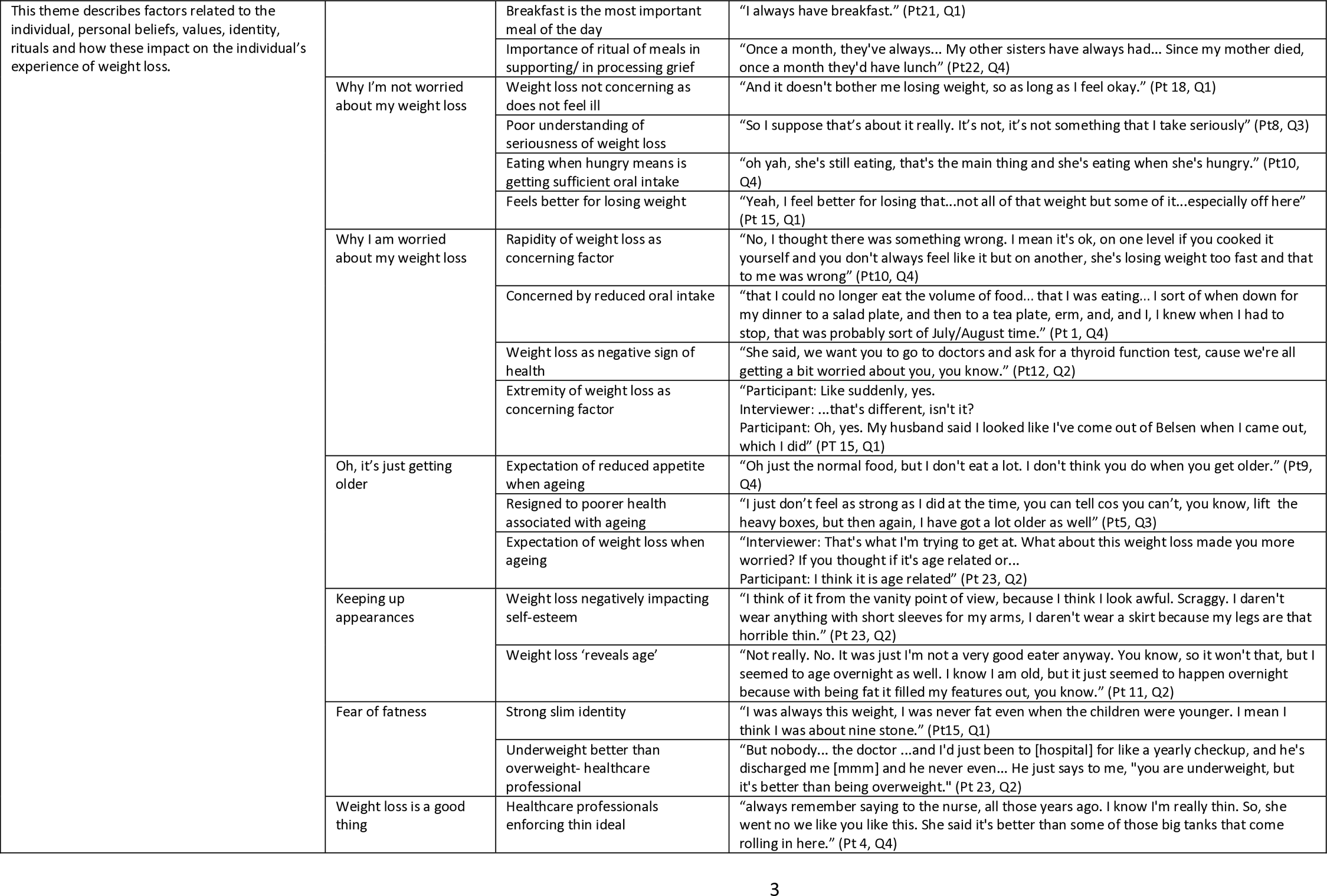

Supplement: S1 Table — (ZIP) [file pone.0321313.s001.zip › PACE Corrected/S1 table.tif]

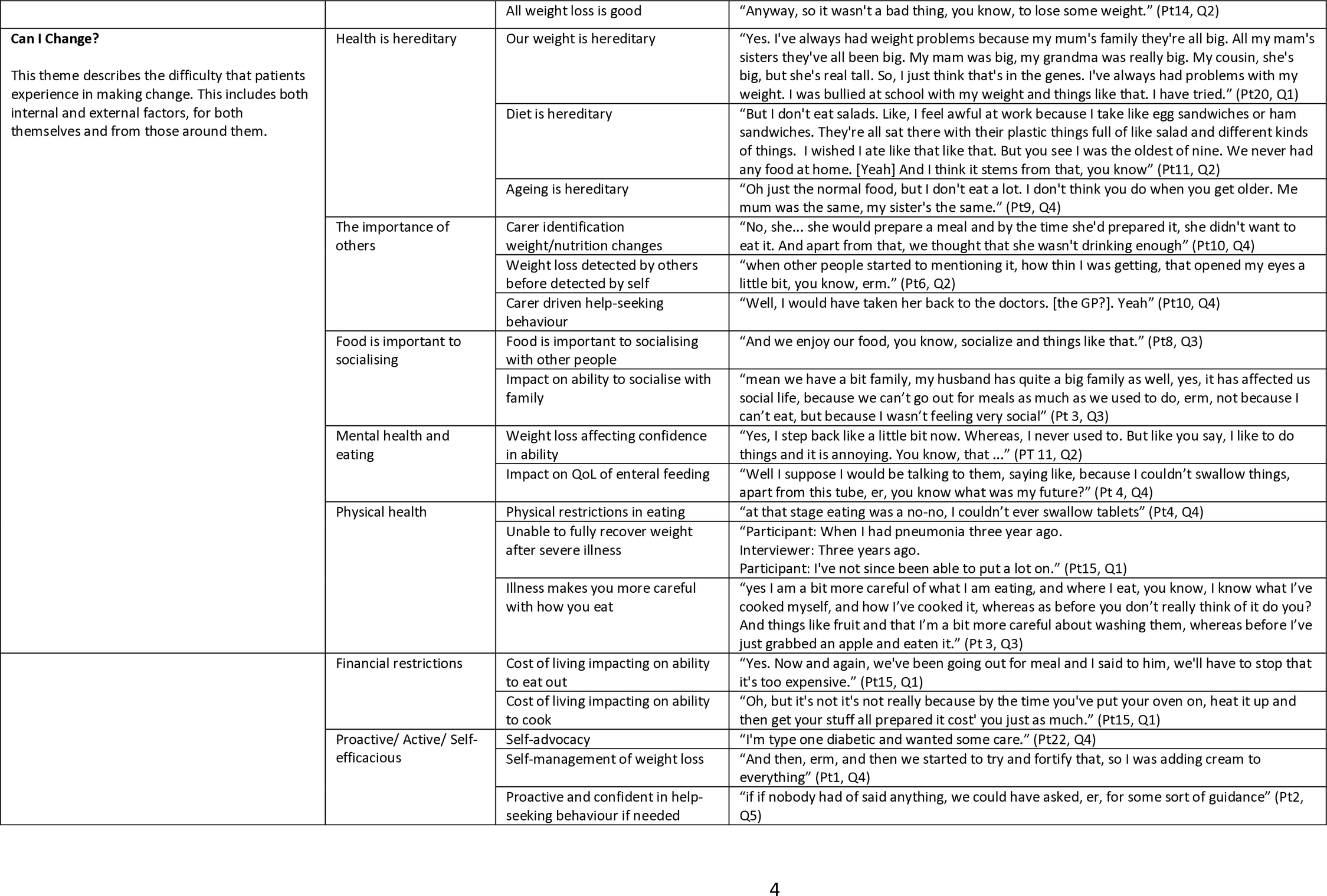

Supplement: S1 Table — (ZIP) [file pone.0321313.s001.zip › PACE Corrected/S1 table.tif]

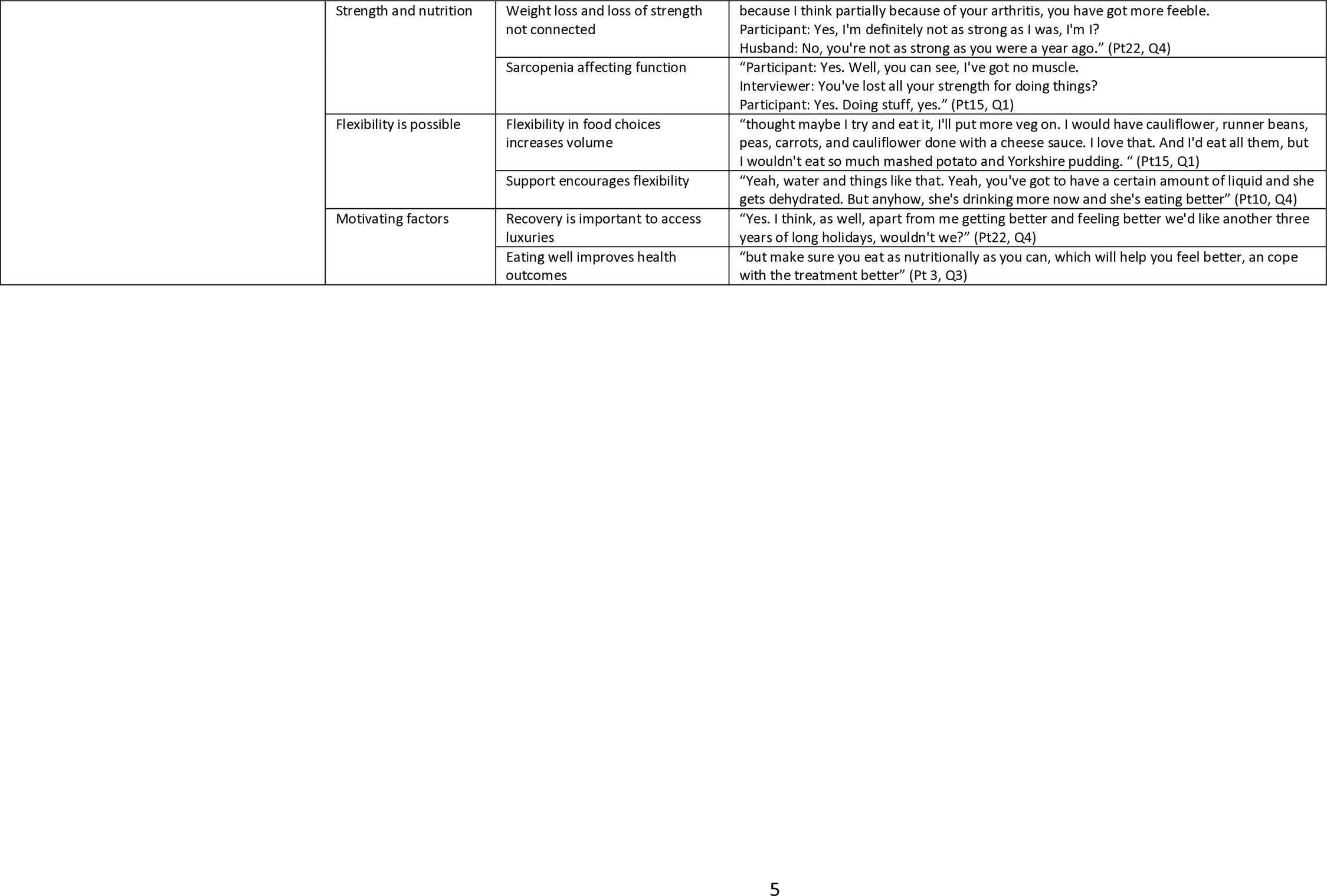

Supplement: S1 Table — (ZIP) [file pone.0321313.s001.zip › PACE Corrected/S1 table.tif]

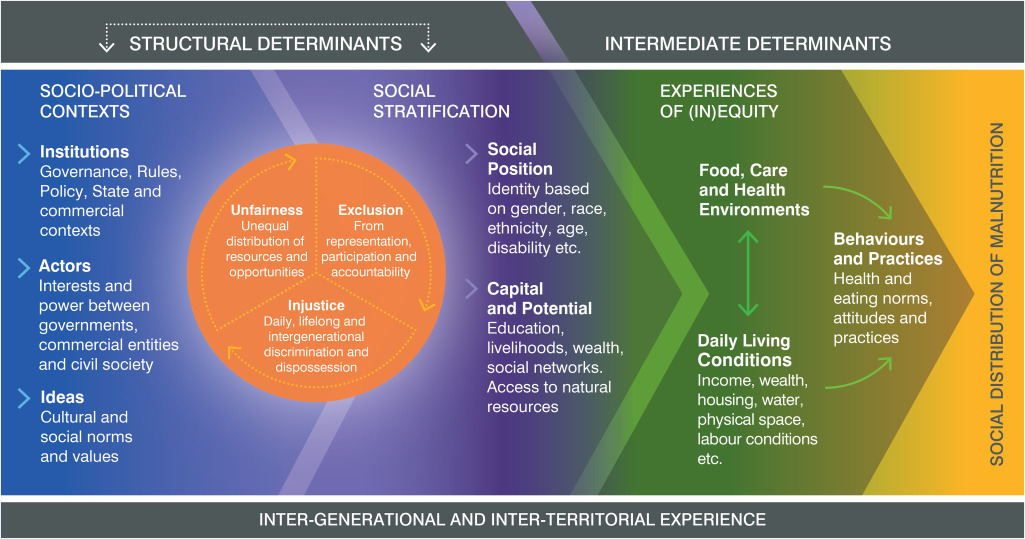

Supplement: S1 Fig — (TIF) [file pone.0321313.s002.tif]
