# Supplementary material for: Socioeconomic status and older adult’s experiences of weight loss: a qualitative secondary analysis
Source: PLoS One. 2025 Apr 22;20(4):e0321313. doi: 10.1371/journal.pone.0321313 (PMC12013945; doi:10.1371/journal.pone.0321313)
Supplement: Attachment — Submitted filename: PONE-D-23-16906_reviewer.pdf [file pone.0321313.s003.pdf]

PLOS ONE

Socioeconomic status and older adult’s experiences of weight loss: a qualitative secondary analysis

--Manuscript Draft--

**Manuscript Number:**	PONE-D-23-16906
**Article Type:**	Research Article
**Full Title:**	Socioeconomic status and older adult’s experiences of weight loss: a qualitative secondary analysis
**Short Title:**	Socioeconomic status and older adult’s experiences of weight loss
**Corresponding Author:**	Anna Newton-Clarke<br>Wolfson Research Institute<br>Hull, --- Select One --- UNITED KINGDOM
**Keywords:**	Nutrition; Geriatrics; weight loss; Socioeconomic status
**Abstract:**	Background: Unintentional weight loss in older adults is common, with 15-20% of those aged >65 having clinically significant weight loss, associated with increased mortality and morbidity. People with socioeconomic disadvantage are more likely to be overweight but also to become frailer in older age. We explore if socioeconomic status impacts upon patients’ experience of unplanned weight loss.<br>Methods: Qualitative secondary analysis of 23 semi-structured interviews with older adults from two prior studies i), those at risk of frailty ii) those with cancer. Reflexive thematic analysis was conducted, using the lens of the Nutrition Equity Framework, on anonymised transcripts with formation of themes and subthemes, with relationships between themes investigated.<br>Results: Mean age 73 years, range 65-87; 34% male, Index of Multiple Deprivation Quintiles IMD 1 (n=9), IMD 2 (n=4), IMD 3 (n=3), IMD 4 (n=6), IMD 5 (n=1). Three major themes were identified. 1. ‘Healthcare Systems’; interactions with either public health or individual healthcare systems influence patient experiences of weight loss. 2. ‘Personal Factors’; that influence a patient’s view of weight loss and the likelihood of weight loss prompting help-seeking behaviour 3. ‘Can I Change?’; patients’ perspectives of their ability to implement change. Factors in each of the themes were understood through motivating (reinforcing) and demotivating (balancing) factors.<br>Conclusion: This study demonstrates that there is structural and individual inequity in individual views, identification, and clinical management of weight loss. The consequences of this disproportionately affect the most deprived, further confounding the inequalities that already exist
**Order of Authors:**	Anna Newton-Clarke, MBChB
	Alex Bullock, PhD
	Miriam Johnson, MD, FRCP, MRCP, MBChB(hons)
	Ugochinyere Nwulu, PhD
	Fliss Murtagh, PhD; FRCP; MRCGP; MBBS
**Additional Information:**	
**Question**	**Response**
**Financial Disclosure**<br><br>Enter a financial disclosure statement that describes the sources of funding for the work included in this submission. Review the submission guidelines for detailed requirements. View published research articles from *PLOS ONE* for specific examples.	The author(s) received no specific funding for this work

Powered by Editorial Manager® and ProduXion Manager® from Aries Systems Corporation

This statement is required for submission and **will appear in the published article** if the submission is accepted. Please make sure it is accurate.<br><br>**Unfunded studies**<br>Enter: *The author(s) received no specific funding for this work.*<br><br>**Funded studies**<br>Enter a statement with the following details:<br>• Initials of the authors who received each award<br>• Grant numbers awarded to each author<br>• The full name of each funder<br>• URL of each funder website<br>• Did the sponsors or funders play any role in the study design, data collection and analysis, decision to publish, or preparation of the manuscript?<br>• **NO** - Include this sentence at the end of your statement: *The funders had no role in study design, data collection and analysis, decision to publish, or preparation of the manuscript.*<br>• **YES** - Specify the role(s) played.<br><br>* typeset	
**Competing Interests**<br><br>Use the instructions below to enter a competing interest statement for this submission. On behalf of all authors, disclose any competing interests that could be perceived to bias this work—acknowledging all financial support and any other relevant financial or non-financial competing interests.<br><br>This statement is required for submission and **will appear in the published article** if the submission is accepted. Please make sure it is accurate and that any funding sources listed in your Funding Information later in the submission form are also declared in your Financial Disclosure statement.<br><br>View published research articles from	I have read the journal's policy and the authors of this manuscript have the following competing interests: Fliss Murtagh is a National Institute for Health and Care Research (NIHR) Senior Investigator. The<br>views expressed in this article are those of the author(s) and not necessarily those of the NIHR, or<br>the Department of Health and Social Care.

Powered by Editorial Manager® and ProduXion Manager® from Aries Systems Corporation

PLOS ONE for specific examples.<br><br>**NO authors have competing interests**<br><br>Enter: *The authors have declared that no competing interests exist.*<br><br>**Authors with competing interests**<br><br>Enter competing interest details beginning with this statement:<br><br>*I have read the journal's policy and the authors of this manuscript have the following competing interests: [insert competing interests here]*<br><br>* typeset	
**Ethics Statement**<br><br>Enter an ethics statement for this submission. This statement is required if the study involved:<br><br>• Human participants<br>• Human specimens or tissue<br>• Vertebrate animals or cephalopods<br>• Vertebrate embryos or tissues<br>• Field research<br><br>Write "N/A" if the submission does not require an ethics statement.<br><br>General guidance is provided below. Consult the submission guidelines for detailed instructions. **Make sure that all information entered here is included in the Methods section of the manuscript.**	Both studies were approved by the NHS Ethics Committee (reference 19/LO/1479, reference 18/YH/0470), with studies performed in accordance with ethical standards laid down in the 1964 Declaration of Helsinki and its later amendments, with informed consent, including consent for secondary analysis, obtained from all participants. For this secondary analysis, authors had access to anonymised data that could not identify participants. All participants gave informed written consent which included the use of their anonymised data for future research by authorised researchers. Original data was collected during the years 2019-2021 as part of two PhD projects.

Powered by Editorial Manager® and ProduXion Manager® from Aries Systems Corporation

**Format for specific study types**<br><br>**Human Subject Research (involving human participants and/or tissue)**<br>• Give the name of the institutional review board or ethics committee that approved the study<br>• Include the approval number and/or a statement indicating approval of this research<br>• Indicate the form of consent obtained (written/oral) or the reason that consent was not obtained (e.g. the data were analyzed anonymously)<br><br>**Animal Research (involving vertebrate animals, embryos or tissues)**<br>• Provide the name of the Institutional Animal Care and Use Committee (IACUC) or other relevant ethics board that reviewed the study protocol, and indicate whether they approved this research or granted a formal waiver of ethical approval<br>• Include an approval number if one was obtained<br>• If the study involved *non-human primates*, add *additional details* about animal welfare and steps taken to ameliorate suffering<br>• If anesthesia, euthanasia, or any kind of animal sacrifice is part of the study, include briefly which substances and/or methods were applied<br><br>**Field Research**<br><br>Include the following details if this study involves the collection of plant, animal, or other materials from a natural setting:<br>• Field permit number<br>• Name of the institution or relevant body that granted permission	
**Data Availability**<br><br>Authors are required to make all data underlying the findings described fully available, without restriction, and from the time of publication. PLOS allows rare exceptions to address legal and ethical concerns. See the PLOS Data Policy and FAQ for detailed information.	Yes - all data are fully available without restriction

A Data Availability Statement describing where the data can be found is required at submission. Your answers to this question constitute the Data Availability Statement and **will be published in the article**, if accepted.<br><br>**Important:** Stating 'data available on request from the author' is not sufficient. If your data are only available upon request, select 'No' for the first question and explain your exceptional situation in the text box.<br><br>Do the authors confirm that all data underlying the findings described in their manuscript are fully available without restriction?	
**Describe where the data may be found in full sentences. If you are copying our sample text, replace any instances of XXX with the appropriate details.**<br><br>• If the data are **held or will be held in a public repository**, include URLs, accession numbers or DOIs. If this information will only be available after acceptance, indicate this by ticking the box below. For example: *All XXX files are available from the XXX database (accession number(s) XXX, XXX.).*<br>• If the data are all contained **within the manuscript and/or Supporting Information files**, enter the following: *All relevant data are within the manuscript and its Supporting Information files.*<br>• If neither of these applies but you are able to provide **details of access elsewhere**, with or without limitations, please do so. For example:<br><br>*Data cannot be shared publicly because of [XXX]. Data are available from the XXX Institutional Data Access / Ethics Committee (contact via XXX) for researchers who meet the criteria for access to confidential data.*<br><br>*The data underlying the results presented in the study are available from (include the name of the third party*	All relevant data are within the manuscript and its Supporting Information files

and contact information or URL).<br>• This text is appropriate if the data are owned by a third party and authors do not have permission to share the data.<br><br>* typeset	
Additional data availability information:	

Manuscript

Click here to access/download;Manuscript;submission 20.06.23.docx

Socioeconomic status and older adult's experiences of weight loss: a qualitative secondary analysis

Authors

Anna Newton-Clarke, Academic Foundation Doctor, Yorkshire and Humber Health Education England, Wolfson Palliative Care Research Centre, Hull York Medical School, University of Hull, Hull

Miriam J Johnson, Professor of Palliative Medicine, Wolfson Palliative Care Research Centre, Hull York Medical School, University of Hull, Hull

Ugochinyere Nwulu, Wolfson Palliative Care Research Centre, Hull York Medical School, University of Hull, Hull

Fliss EM Murtagh, Professor of Palliative Care, Wolfson Palliative Care Research Centre, Hull York Medical School, University of Hull, Hull

Alex F Bullock, Research Associate, Wolfson Palliative Care Research Centre, Hull York Medical School, University of Hull, Hull

ABSTRACT

Objectives: Unintentional weight loss in older adults is common, with 15-20% of those aged >65 having clinically significant weight loss, associated with increased mortality and morbidity. People with socioeconomic disadvantage are more likely to be overweight but also to become frailer in older age. We explore if socioeconomic status impacts upon patients' experience of unplanned weight loss.

Methods: Qualitative secondary analysis of 23 semi-structured interviews with older adults from two prior studies i), those at risk of frailty ii) those with cancer. Reflexive thematic analysis was conducted, using the lens of the Nutrition Equity Framework, on anonymised transcripts with formation of themes and subthemes, with relationships between themes investigated.

Results: Mean age 73 years, range 65-87; 34% male, Index of Multiple Deprivation Quintiles IMD 1 (n=9), IMD 2 (n=4), IMD 3 (n=3), IMD 4 (n=6), IMD 5 (n=1). Three major themes were identified. 1. 'Healthcare Systems'; interactions with either public health or individual healthcare systems influence patient experiences of weight loss. 2. 'Personal Factors'; that influence a patient's view of weight loss and the likelihood of weight loss prompting help-seeking behaviour 3. 'Can I Change?'; patients' perspectives of their ability to implement change. Factors in each of the themes were understood through motivating (reinforcing) and demotivating (balancing) factors.

Conclusions: This study demonstrates that there is structural and individual inequity in individual views, identification, and clinical management of weight loss. The consequences of this disproportionately affect the most deprived, further confounding the inequalities that already exist.

Key messages

What is already known on this topic – It is already known that weight loss has negative effects on morbidity and mortality in older adults. It is also known that socioeconomic status influences weight throughout life.

What this study adds – This study adds evidence of the impact on socioeconomic status on older adult's views of unintentional weight loss. This is explored through differing perceptions of diet, public health messages and interactions with healthcare professionals. Older adults from more deprived areas are more likely to feel stigma surrounding their diet, less likely to be flexible in their dietary patterns and less likely to advocate for themselves in interactions with healthcare professionals.

How this study might affect research, practice or policy – This study advocates for more research on how best to change eating practices in older age to combat weight loss and accelerated sarcopenia. It provides evidence for the negative impact of public health messages on weight loss in older adults and suggests that to positively change weight loss in older adults, we must remove food guilt and tailor dietary advice to an individual's circumstances.

INTRODUCTION:

Clinically significant unintentional weight loss in older adults is a component of frailty (1). Weight loss is common (15-20% ≥65 years), especially in vulnerable groups such as nursing home residents, and is associated with increased mortality, morbidity, and reduced quality of life (2, 3).

In England, public health expenditure on malnutrition and its effects in 2011-12 was estimated to be £19.6 billion, more than 15% of the total spending on health and social care, over half of which was in older adults (4). Public health messages, including "thin-ness" as desirable, following high fibre, low-energy-dense diet, and discouragement of snacking are often considered applicable to older adults, and may lead to dietary restrictions (5, 6)

However, weight loss in the older adult, without protein supplementation and appropriate graded exercise, can lead to loss of muscle mass in addition to fat mass, potentially accelerating sarcopenia (7) This is particularly important in the older person, who may disproportionately lose functionality and capacity for independent living (7). Despite this, healthcare professionals and patients often believe weight loss is a positive or expected part of ageing (8). Malnutrition is a condition that primary care health professionals have reported feeling unsupported and unknowledgeable about managing, with services for obese and overweight adults prioritised over management malnutrition (8).

We also know that socioeconomic status (SES) is associated with weight (9). More affluent older adults often have a more favourable BMI (body mass index) and weight circumference profile (9), with disadvantaged persons more likely to be overweight in adult life, but also to become frailer in older age (9). Poorer SES is associated with accelerated decline in older adults in multiple domains of health, independently of diagnosed health conditions (10). Within the complex and confounding factors of SES, age and weight loss, qualitative research may allow a greater understanding of individual experience. The aim of this study is to explore how SES relates to how older adults, with various medical conditions and across different SES, view their unintentional weight loss.

METHODS:

This is a secondary analysis of 23 semi-structured interview transcripts. Interviews were conducted in two studies looking at nutrition and weight loss in older adults. Interviews were undertaken by AB and UN with older adults, defined as 65 or over. Interviews were transcribed verbatim and anonymised. Transcripts were analysed as a single dataset.

We used a pragmatic approach, focusing on real-life clinical application and analysed the data following the six phases of reflexive thematic analysis (11). Familiarisation and initial note taking was conducted by ANC who coded an initial set of 25% of the interviews in a data-driven, inductive approach. These were then independently coded by two members of the research team (AB, MJ). Discrepancies in coding were discussed with the intent of enriching analysis through collaboration, prior to the formation of a codebook. ANC coded the remainder of the dataset, forming both semantic and latent codes using constant comparison. Codes were grouped together to form subthemes and then organised into descriptive themes, each unified by a single, central concept. These candidate themes were discussed and refined (AB, MJ, ANC) to form analytic themes. See online supplementary **Table 1** for themes table. Once the themes had been finalised, participant, area-based SES was applied, using the Index of Multiple Deprivation (based on the 2019 Census and postcode (12), Quintile 1 (highest), to 5 (lowest). This allowed for the data to be understood in the

light of how participants with different SES related to subthemes, creating a depth and richness in the analysis. SES was applied to the data at this point to minimise any bias that might have been present if this was added before analysing the data. In addition, we applied the lens of the Nutrition Equity Framework to contextualise the participant's lived experience within the wider, structural inequity of nutrition (13).

This framework identifies how social and political structures interact and shape inequity in nutrition, looking at unfairness, exclusion and injustice understood through unequal power dynamics. Understanding how these factors interact within the wider socio-political contexts is important when implementing long-term and sustainable changes. We adapted and simplified this framework to make sense of study results, focusing on the intermediate determinants of nutrition and how this results in individual factors, with an appreciation for the contextual driver of the structural determinants.

Author reflexivity, important to the interaction between researcher and data, was completed throughout the analysis: ANC is university-level educated medical clinician, within two years of qualification. MJ is a senior applied health researcher with 38 years as a medical clinician, mostly within the specialty of palliative care. AB is a clinical academic dietitian.

Both studies were approved by the NHS Ethics Committee (reference 19/LO/1479, reference 18/YH/0470), with studies performed in accordance with ethical standards laid down in the 1964 Declaration of Helsinki and its later amendments, with informed consent, including consent for secondary analysis, obtained from all participants. For this secondary analysis, authors had access to anonymised data that could not identify participants. All participants gave informed written consent which included the use of their anonymised data for future research by authorised researchers. Original data was collected during the years 2019-2021 as part of two PhD projects.

RESULTS:

Twenty-three semi-structured interview transcripts were analysed (mean age 73yrs, range 65 to 87; 34% men; 35% cancer diagnosis; 64% at risk of frailty). 3 themes were generated from the data; 1) Interaction between healthcare systems and the individual, 2) Personal factors impacting on an individual's view of their weight loss, and 3) Can I Change?

Theme 1: Interactions between healthcare systems and the individual:

All participants expressed the public health message of healthy eating, implicitly or explicitly appraising the value of their diet against this. Participants living in less deprived areas discussed these more positively, viewing their own diet as in line with the public health messages. This was reflected in a feeling of capability and empowerment in their diet.

> "*Other than… erm… I we've always had skimmed milk, hah, as that's more healthy, but [Dietitians recommend], full fat milk, so will have to look at that, but other than that I seem to be, [Dietitian] seem to be thinking I was doing all the right things really" (Participant 2, Quintile 5)"*

> *"I always have a lot of fruit and veg, and things like that so, it hasn't been hard for me at all" (Participant 3, Quintile 3)*

However, participants living in more deprived areas viewed their own diets negatively, as they did not conform with expected ideals.

> *"But I don't eat salads. Like, I feel awful at work because I take like egg sandwiches or ham sandwiches. They're all sat there with their plastic things full of like salad and different kinds of things. I wished I ate like that like that" (Participant 12, Quintile 2)*

Some participants felt stigmatised and judged negatively by healthcare providers about their weight and felt burdened by the language of these interactions.

> *"You've got to be careful on how you phrase it because there are some that cannot lose [weight] and they're feeling picked on. You know, it's not right"(Participant 21, Quintile 1)*

Conversely, others felt healthcare professionals did not act on their concerns about weight loss, with healthcare professionals felt to minimise nutritional issues leaving participants feeling abandoned.

> *"He just says to me, "you are underweight, but it's better than being overweight." They don't seem bothered about my weight and yet..." (Participant 23, Quintile 2)*

> *"Yeah, that's what I feel like. Like before. I felt a bit abandoned, you know, because it's like there's nothing the matter. So, go home. Then you go home, and you think but why am I still getting these pains in my stomach? You know, why not something probably like the said the IBS [irritable bowel syndrome], but I get really a lot of pain in my back, you know at the bottom of my ribs coming around into my chest like that all the time" (Participant 12, Quintile 2)*

The ability to challenge this attitude and facilitate change was more readily expressed by participants in less deprived areas, who persevered until a better outcome was forthcoming.

> *"The only frustration was this weight loss, lac of energy started to arise, just getting somebody to take it onboard which has now happened, and we have seen the improvement, but the period, I suppose between end of September through to… beginning of this year when I felt a little frustrated because we raised these issues… and we… were… not fobbed-off that's too strong a word, but nothing really materialised" (Participant 1, Quintile 4)*

Where positive interactions with healthcare professionals regarding weight loss were expressed, participants were more often living in less deprived areas, and expressed trust with their healthcare professionals, stating confidence that they would act on their behalf.

> *"I think I told them, … [NAME] was my personal nurse, and we poured our heart out to [her] …" (Participant 4, Quintile 4)*

> *"No my doctor, my ordinary doctor… Well I told him. I wasn't eating! [laugh] I said I'm kinda losing weight, I'm not eating, ha, yeah, and this is why, this is why he put me for the scan, because, it got to the stage where I wasn't eating solid food" (Participant 5, Quintile 3)*

Interactions with healthcare professionals were described more passively by those in more deprived areas, whereby participants experienced interventions as happening to them, rather than being self-driven:

> *"She checked it on the computer. That's why she says I'm putting you... I told you I wasn't keen on them other foods, they're in there on the kitchen, whatever they call them. She said, I'll send you a sample pack and I got those and I got me drinks" (Participant 13, Quintile 1)*

Theme 2: Personal factors impacting on an individual's view of their weight loss:

Subtheme 1: Rituals and Routines

Rituals of eating and the traditional 'three meals a day' eating pattern was shared by all participants although language used to describe this differed. This was portrayed as a rigid and inflexible pattern, sometimes reinforced by healthcare professionals.

> *"If we're just on our own we have a cup of tea and a biscuit. At lunchtime we have sandwiches and then we have a cooked meal of an evening" (Participant 9, Quintile 4)*

> *"But he [Doctor] said, " you must have a regular mealtime" which I do. I'll have a onc..." (Participant 15, Quintile 1)*

This routine was particularly expressed by those in more deprived areas, especially highlighting the importance of Sunday lunch. This tradition was maintained despite challenges e.g., difficulty cooking or reduced appetite. The ritual of the meal appeared to be almost more important than the eating itself, with strong social implications and family ties.

> *Participant: I get breakfast in, when me daughters aren't here. Yeah, I get me Sunday lunch delivered [right] which I pay for but it's very enjoyable" (Participant 17, Quintile 1)*

> *"I can cook myself a Sunday dinner and have two mouthfuls and I'll throw it" (Participant 20, Quintile 1)*

> *"… I often go [to grandsons] weekend, like a Sunday for me Sunday lunch" (Participant 15, Quintile 1)*

Food was described by all participants to socialising, both amongst family and with friends, for celebrations and grief, forming a space in which to maintain these relationships in changing circumstances.

> *"Once a month, they've always... My other sisters have always had... Since my mother died, once a month they'd have lunch" (Participant 22, Quintile 4)*

> *"And we enjoy our food, you know, socialize and things like that" (Participant 8, Quintile 3)*

> *"I mean we have a big family, my husband has quite a big family as well, yes, it has affected us social life, because we can't go out for meals as much as we used to do, erm, not because I can't eat, but because I wasn't feeling very social" (Participant 3, Quintile 3)*

> *"May this year, me and [wife] 44th wedding anniversary and we all went for, for, a family meal - well, they had a family meal, I just had a beer" (Participant 17, Quintile 1)*

Subtheme 2: Why I am not worried about my weight loss

Justifications for not being worried about weight loss were complex, these included; being slim throughout adult life, never being particularly interested in food, or not feeling unwell, lead to reduced concern about weight loss in older age.

> *"Participant: I lost a stone… I've always been on the slim side" (Participant 16, Quintile 1)*

> *Not really. No. It was just I'm not a very good eater anyway. You know, so it won't that, but I seemed to age overnight as well" (Participant 9, Quintile 4)*

"Oh no, no, no it doesn't play on me mind, no but it could do if you were that type of person" (Participant 5, Quintile 3)

Effect of stress, grief, and the burden of caring for a loved one, was also associated with weight loss. The link with weight loss did require initial coaxing from the interviewer, and seen as was less concerning, as it was seen as having a causative reason.

"Well, I started losing weight because I'd been looking after my husband, who was poorly. I started losing the weight and then he died and I still kept losing the weight. I used to be about 10"10'... I'm seven stone something now and I cannot put it on" (Participant 23, Quintile 2)

"Interviewer: I mean, when did your husband die?...

Participant: Four years ago, August… I think that had something to do with it" ((Participant 16, Quintile 1)

From those living in more deprived areas, this normalisation of changing food habits was discussed in comparison to others, either other people of the same age or in relation to how family members were as they aged:

"Oh just the normal food, but I don't eat a lot. I don't think you do when you get older" (Participant 9, Quintile 4)

"Eating like...I have my dinner, say... I don't have as much dinner as I did, but I think as you get older, everybody is, because you talk to people in here [hospital ward] and they always say they don't eat as much because you're not moving about, you're not at work" (Participant 23, Quintile 2)

Eating something, even if less than previously, was considered to be better than eating nothing and helped minimise anxiety. Participants also compared their experience favourably to others, feeling grateful for what they have.

"And I see people who're properly poorly like my daughter died of motor neurone disease. And then nine and a half months later my husband died of it as well. So, I have seen stuff and I've seen stuff on the world and when I worked for [private hospital], I've seen stuff there and that is horrendous. And I think just thank your lucky stars, but I still go "Oh my stomach!", you know what I mean and then I think "shut up!"" (Participant 12, Quintile 2)

"We've seen a lot of other people who are a lot more poorly than we are. And the more you come here, there more you listen and think, crikey, I have nothing to worry about, you hear what other have, and there still here and they're still fighting, leading their life as normal as possible" (Participant 3, Quintile 3)

Some participants were pleased to lose weight, albeit unintentionally. Some were so pleased that they hoped to continue the weight loss intentionally. Whilst others expressed a desire to regain weight.

"I'm not bothered about it, not really. It's a good thing to lose it, I'm not saying... that... but I'm not concerned" (Participant 14, Quintile 2)

"I like it, I like being a bit more slimmer. I'm not skinny, skinny, but I could do with putting a little bit more, but I don't want to put too much on" (Participant 15, Quintile 1)

__Husband__: "You are about where you should be now in weight terms…
__Participant__: I have been trying to lose weight for 50 years… I'd like to lose some more weight in the next... this month?" (Participant 22, Quintile 4)

Participants, particularly living in more deprived areas, having previously been overweight, were used to being actively encouraged to lose weight by healthcare professionals. Being overweight in this context was experienced as a barrier to accessing healthcare services.

"So, I said right I'll go on a diet. So, I stuck to the diet every bit I stuck to the diet. He [GP] gave me a sheet, thousand calories a day" (Participant 18, Quintile 1)

__Participant: "__Well, it's a little different now because I went to see this physiotherapist and she didn't even weigh me or measure me. She said, "How tall are you?" I said, "5ft 4." "How much do you weigh?" I told her, I said, "15 2." She said, "Oh, your BMI is 36 you've got to have it under, you've got to lose weight.
__Interviewer:__ For the surgery?
__Participant:__ No, just to see the specialist" (Participant 20, Quintile 1)

For all participants, there was a resignation to a loss of appetite and lower level of health associated with ageing.

"Resigned? You can't help but be resigned, I mean, I can't do the things that I used to do now, I'm reconciled to it" (Participant 8, Quintile 3)

"I mean, I get tired but I think that's old age. I don't think that's anything to do with my health. As I say, I've nothing to complain about" (Participant 23, Quintile 2)

Subtheme 3: Why I am worried about my weight loss

In general, concerns arose only with a very noticeable, and rapid, change in appearance or feeling ill. Rapid weight loss was viewed as concerning amongst all participants, stimulating help-seeking behaviour, and a reason to seek medical assistance.

"No it was the weight loss love, yeah. I didn't, I didn't realise how much I was losing, and how quickly… it didn't, it didn't strike me at first, and then I realised, then I thought you know this is too much, I've got to do something an I went to the doctors…" (Participant 6, Quintile 1)

"No, I thought there was something wrong. … she's losing weight too fast and that to me was wrong… half of her clothes didn't fit her" (Participant 10 and Carer of Participant 9, Quintile 4)

"Started at nine stone three, and, and then this seven started appearing, and I'm thinking this really isn't good news, you know…" (Participant 1, Quintile 4)

"Well, only if it did drop very drastically, then I'd know there's something wrong. It's coming off bit by bit, so I know... [it's nothing to worry about]... but if I get up tomorrow and I'm about five stone then I'd think, "Oh my God, what's wrong?"" (Participant 21, Quintile 1)

Face to face consultations were preferred over telephone consultations; allowing for physical cues to action to be seen more easily.

"You see the problem is you can't go in to see your GP to really explain it or show him how you're losing weight, so I suppose he's at a disadvantage, so there's everything against it really, as far as that bit goes" (Participant 5, Quintile 3)

When he [doctor] lifted my top, he could see how much weight I'd lost, and my clothes was hanging down. Anyway, he went "oh goodness gracious!". I mean, he felt down the side of my stomach and it hurt, and he went. "Oh, I'm sending you to a hospital for x-rays" (Participant 12, Quintile 2)

In addition to weight loss, visible reduction in oral intake was another cause for concern, although mainly for participants in higher deciles.

"…that I could no longer eat the volume of food… that I was eating… I sort of when down for my dinner to a salad plate, and then to a tea plate..." (Participant 1, Quintile 4)

Subtheme 4: Weight loss and the importance of appetite

Participants perception of appetite and its relationship to their diet and weight varied. Some participants expressed an understanding of the importance of appetite in weight loss:

"But I was really pleased, it was only a bit recently, when I was able to get suddenly started to get my appetite back" (Participant 2, Quintile 5)

Although some participants in more deprived areas expressed difficulty in understanding why they were losing weight when there was sufficient food available, lacking an appreciation for the importance of appetite in being motivated to eat.

***Husband interjects**: "She has cupboard full of food and she won't touch it" (Participant 18, Quintile 1)*

"I don't know. I just cook it and just love nothing. I don't want this" (Participant 16, Quintile 1)

Going to a restaurant for a meal was seen to have a positive effect on appetite, however for participants in more deprived areas there was a fear of food waste, which impacted on this effect:

"Yeah, I would eat it, but I would leave some of it Yeah. You know, when I go for a meal, I put on what I think that I can eat" (Participant 15, Quintile 1)

Theme 3: Can I Change?

Subtheme 1: Patient perceptions of barriers to change

A lack of flexibility in dietary patterns restricted participants ability to make changes in their eating. A determination to maintain normality despite needing to make changes was seen, with a feeling of frustration at lack of knowledge on how to gain weight, and perseverance of large portion sizes despite finding them off putting.

"No, not really. I was losing weight and I couldn't put weight [on], and I still can't put weight on. No matter what I eat, I cannot put weight on. I don't worry about it now. I won't say I'm a worrier, I'm not, but I just think about it - "I wish I could put some weight on" (Participant 23, Quintile 2)

"No not really, just keep as normal as you had before" (Participant 3, Quintile 3)

Participants living in more deprived areas particularly expressed difficulty with portion sizes, this was reinforced by negative comments regarding wasting food from relatives.

"Oh no, it's just the way I eat has changed and portions have changed. I don't... I can't eat it if I have a plate loaded with food. I can't eat that"… "If I put too much on, he goes, "Your eyes are bigger than your belly" sort of thing" *(Participant 15, Quintile 1)*

"I just can't get it down me. I feel sick, not sick but I said, "I'm full." (Participant 13, Quintile 1)

This lack of flexibility also placed the emphasis on ensuring that participants had at least one, hot, square meal a day. This was seen as more important that the amount consumed in its entirety.

"But, we always make sure that my mam has a full meal one day. There's always food there, whatever. We'll go out. I go out on a Saturday with my mam, basically every weekend for the last two or three years, we always have a full meal and whatever" (Son of Participant 13, Quintile 1)

A sense of disengagement was felt with weight changes as participants aged, as it was felt to be predetermined by the patterns set by their family members before them.

"Yes. I've always had weight problems because my mum's family they're all big. All my mam's sisters they've all been big. My mam was big, my grandma was really big. My cousin, she's big, but she's real tall. So, I just think that's in the genes. I've always had problems with my weight. I was bullied at school with my weight and things like that. I have tried" (Participant 20, Quintile 1)

There was a sense, particularly for those in more deprived areas, of food guilt. This was founded in participants trying and failing to conform to the socially normal eating patterns. This was also felt in guilt of being unable to eat as 'healthily' as others around them, attributed to food poverty as a child.

"I don't eat breakfast, I don't eat lunch, and sometimes for my tea I'll just have some toast or a sandwich. I know it's wrong" (Participant 20, Quintile 1)

"But you see I was the oldest of nine. We never had any food at home. [Yeah] And I think it stems from that, you know" (Participant 12, Quintile 2)

Those in more deprived areas also expressed a belief that they deserved ill health in older age due to a lifetime of not eating as they should:

"I did. And when [Doctor] came to see me, he looked at me and when "Huh" and I went "I know!" and I said it's a lifetime of a bad diet. It's my own fault" (Participant 12, Quintile 2)

Whilst some shared a feeling of disillusionment with public health messages, finding them inaccessible and off-putting:

"They're saying you shouldn't eat meat. You shouldn't eat this. Might just as well give up" (Participant 21, Quintile 1)

Some of the challenges of eating in older age were affected by loss of independence. This was mainly expressed by those living in more deprived areas. Independence involved both being able to feed oneself and the financial independence to be able to afford to cook and dine out.

Yes. Now and again, we've been going out for meal and I said to him, we'll have to stop that it's too expensive" (Participant 15, Quintile 1)

"I use to make my own [meals] but it's not worth it for the money" (Participant 13, Quintile 1)

Independence was also affected by confidence. Lack of confidence impacted on activities that were once taken for granted and now required input from others; such as cooking and to be cared for.

"No, well, I maybe warm some soup up. The thing is, it's only this last month or more I'm a bit dithery. And [son] don't, [son] would simply come and sort dinner" (Participant 13, Quintile 1)

Both physical and mental health impacted on participants ability to eat. This ranged from physical health conditions affecting ability to chew, to changes in cognition affecting participants ability to remember their own weight loss.

"And me teeth...? I couldn't keep them in, with false teeth which just fell out because I've lost that much weight. Round my mouth and my face, me teeth were too big for me mouth" (Participant 15, Quintile 1)

"Yeah, we mentioned it on several ...I mentioned it on several occasions so I know I mean, yeah, I know that [patient]'s memory is not so good so he can't remember and I now I've have mentioned it…" (Friend and carer of Participant 11, Quintile 2)

Experiences of ill-health had repercussions for psychological well-being surrounding eating. This was expressed with a loss of spontaneity and freedom with eating and the effect on appetite and motivation to eat.

"Yes I am a bit more careful of what I am eating, and where I eat, you know, I know what I've cooked myself, and how I've cooked it, whereas as before you don't really think of it do you? And things like fruit and that I'm a bit more careful about washing them, whereas before I've just grabbed an apple and eaten it" (Participant 3, Quintile 3)

"Appetite, I had no appetite. I just didn't think I could manage it and then we all came back here with the grandchildren and had a letter from [hospital] and "the surgery was successful, you're now cancer-free". I just cried [I bet] and I started eating again. I've got my appetite now" (Participant 17, Quintile 1)

Subtheme 2: Perceptions and experiences of being able to implement change.

Support of family members and informal carers was instrumental in identifying, implementing, and advocating for change.

"When other people started to mentioning it, how thin I was getting, that opened my eyes a little bit, you know, erm" (Participant 6, Quintile 2)

"Well, I would have taken her back to the doctors. [the GP?]. Yeah" (Participant 10, carer of Participant 9, Quintile 4)

The caring actions of family members often revolved around cooking for participants, adding to the burden of informal caring responsibilities. Some participants living in more deprived areas, utilised the community around them, paying for the delivery of food, and viewed this as cheaper than cooking for themselves. This also enabled participants to feel in control of their food choices, with family preparing preferred foods, with food freedom seen as important.

__Interviewer__: "Your main meal is from them [the neighbour].

__Participant__: Yeah, and I pay her for that. Which I don't mind because it costs me more if I put the oven on." (Participant 15, Quintile 1)

"She's on 12-hour days on tomorrow and she's going to come and take me shopping on Wednesday with me wheelchair" (Participant 17, Quintile 1)

An enjoyment of the physical changes associated with weight gain were expressed by some participants in more deprived areas, reinforced by their family members.

"And the girls see the difference. Last time I went out, cos I go to [place name], they said "Oh you look wonderful, [name]". Cos last time they saw me, I was like death warmed up basically. Eyes were sunken, cheeks were in here. So, that's coming back now" (Participant 17, Quintile 1)

Participants living in less deprived areas shared a belief in patient-centred healthcare, whereby they could initiate and ask for guidance from their healthcare professionals and vocalised the importance of education to facilitate engagement and pro-activeness in health.

"I would have wanted them to really refer her to a hospital consultant" (Participant 10, carer of Participant 9, Quintile 4)

"I think it, it actually does you a lot of good to actually vocalise and make you sort of think through it, we've both been in education both our lives, and I was quite interested from that point of view" (Participant 1, Quintile 4)

Some participants were flexible in their diets, either driving change through self-help measures or accepting that the goalposts of nutrition were different in the context of weight loss.

"He don't eat a lot of...most of your diet is meat and fried stuff. But at least he's eating" (Friend and carer of Participant 11, Quintile 2)

Future goals were important in motivating participants to change their health and diet. These included luxuries such as travelling abroad, functionality such as maintaining muscle mass and access to further treatments in their cancer pathway. These were individual and varied but allowed for a goalpost for participants to work towards.

"Yes. I think, as well, apart from me getting better and feeling better we'd like another three years of long holidays, wouldn't we?" (Participant 22, Quintile 4)

"Oh, no, I don't want to lose me muscle" (Participant 15, Quintile 1)

"But make sure you eat as nutritionally as you can, which will help you feel better, an cope with the treatment better" (Participant 3, Quintile 3)

Application of the Nutrition Equity Framework

Our application of the Nutrition Equity Framework (13) is summarised schematically in **Figure 1.** The structural societal inequity in the way people eat influences their attitudes and behaviours around unintentional weight loss in older age. The consequences disproportionately affect those that are more deprived, further confounding already existing inequities. This perpetuates further with those from a more deprived areas being less likely to engage in health-seeking behaviours and more passive in consultations with healthcare professionals. This is confounded by the knowledge that

doctors have been shown to give more information and more emotional support in a shared decision-making style with patients with a higher SES (14).

Our research shows collusion between healthcare professionals and participants with the view that all weight loss is positive, which is more pronounced among participants from more deprived areas and those who had been overweight in adult life. These factors result in a vicious cycle of increased unfairness, injustice and exclusion of those from more deprived areas

Discussion:

This is the first qualitative study to stratify older adult's experience of nutrition and weight loss by socioeconomic status, allowing us to further understand the barriers in clinical management of weight loss in this population. This study demonstrates structural societal inequity in the way people approach food and diet, which impacts on their attitudes and behaviours around unintentional weight loss.

Our findings are consistent with previous research identifying that messages from adulthood, including concerns about weight gain, are carried forward into old age (5, 6). We have expanded upon this to identify that those living in more deprived areas more likely to be stigmatised regarding their diet, and to self-blame for choices that deviate from public health messages, a concept previously seen in parents in lower SES with regards to their child's diet (15).

Our findings, that those from more deprived areas were more passive in consultations with healthcare professionals, less able to challenge healthcare professionals, and less likely to alter the outcome of the consultation with their concerns, support other findings providing evidence that participants from lower SES are less likely to raise concerns in healthcare consultations, and are less agenda-setting and information-seeking (16).

In the individual experiences of inequity, we have found that behaviours from earlier life impact on experiences of nutrition and weight loss in later life, as previously seen whereby poverty in childhood has adverse impacts on adult health outcomes (17). In our study, participants living in more deprived areas were more influenced by the ritualistic behaviour of eating, being both reluctant to waste food but also being less able to alter their portion sizes.

Clinical and research implications:

Unintentional weight loss is sometimes a clinically important indication of serious illness which is often overlooked by the person and their clinician alike. For older adults with unintentional weight loss, time should be taken to understand their current diet. This involves understanding what they mean by a "good diet", rituals they observe, and changes they feel capable of making. We may need to remove the guilt of eating higher calorie foods and snacking, releasing them from the messages that have been reinforced since childhood. Older people must be given permission to eat as it suits them, removing the rigidity of set meals time and offer solutions, such as using smaller plates to make portions look less off-putting.

Particularly for those living in more deprived areas, we must not stigmatise how they eat, and take time to undo the negative comparisons that they make to others. For all, but particularly for those from higher deciles, we must take care not to collude on weight loss, ensuring an understanding of the differences between intentional and unintentional weight loss and that patient views on weight loss are appropriate.

Patients, especially those living in more deprived areas, may not initiate conversations surrounding weight loss, believing that healthcare providers would mention it if concerned. Weight loss should be considered as a symptom that needs treating in the context of advanced disease or – contrastingly - after negative investigations. We must not assume that weight loss will be self-managed by patients. Giving permission to alter diets can help to introduce flexibility, empowering patients to make sustainable changes in their diet.

Future research should aim to understand the most effective and sustainable changes in diet for older adults and to further understand how language surrounding weight loss and nutrition impacts on individual views

Strengths and limitations

This study provides detailed, in-depth discussion of patient experience surrounding their weight loss. It is one of the few studies to consider the context of socio-economic status in relation to these experiences. Rich data were included in the study, with a diversity of participants (age, sex, SES), generating a wide range of views.

Limitations include the assignment of SES to participant by postcode. This means that SES is by an area-based measure as opposed to an individual measure and may not be entirely representative of individual status. The interviews were initially conducted to answer a different research question; therefore, all the pertinent information may not have been provided during data collection. Data were only collected on a small subset of the population in one geographic area; therefore, the findings may not be universally transferable to the wider population although we anticipate these findings will be applicable beyond our study settings.

Conclusion

The importance of socioeconomic factors in health is increasingly apparent. This study adds to current research patents' perspectives with regards to weight loss and nutrition. Socioeconomic status affects patients views of their own weight loss, how they engage with healthcare professionals and the outcome of these patients bringing forward their concerns. Healthcare providers should consider SES when tackling nutrition, particularly focusing on addressing underlying health beliefs and belief in capacity to change. Care must be taken to undo unhelpful health messages and redefine the goalposts for weight and nutrition in older adults.

Conflict of Interest Statement:

Fliss Murtagh is a National Institute for Health and Care Research (NIHR) Senior Investigator. The views expressed in this article are those of the author(s) and not necessarily those of the NIHR, or the Department of Health and Social Care.

Contributorship statement:

The project was designed by AB and MJ. DC provided overarching support and guidance. Data collection was conducted by AB. Data analysis was conducted by AB, LP, and MJ. ANC wrote the manuscript. All authors revised the manuscript critically. AB and MJ have overall responsibility for the final content.

References

1. Fried LP, Tangen CM, Walston J, Newman AB, Hirsch C, Gottdiener J, et al. Frailty in older adults: evidence for a phenotype. J Gerontol A Biol Sci Med Sci. 2001;56(3):M146-56.
2. Rasheed S, Woods RT. Malnutrition and quality of life in older people: a systematic review and meta-analysis. Ageing Res Rev. 2013;12(2):561-6.
3. Shahar A, Shahar D, Kahar Y, Nitzan-Kalusky D. [Low-weight and weight loss as predictors of morbidity and mortality in old age]. Harefuah. 2005;144(6):443-8, 52.
4. Elia M. The cost of malnutrition in England and potential cost savings from nutritional interventions (short version). Redditch, UK: On behalf of BAPEN 2015.
5. Castro PD, Reynolds CM, Kennelly S, Geraghty AA, Finnigan K, McCullagh L, et al. An investigation of community-dwelling older adults' opinions about their nutritional needs and risk of malnutrition; a scoping review. Clin Nutr. 2021;40(5):2936-45.
6. Avgerinou C, Bhanu C, Walters K, Croker H, Liljas A, Rea J, et al. Exploring the Views and Dietary Practices of Older People at Risk of Malnutrition and Their Carers: A Qualitative Study. Nutrients. 2019;11(6).
7. Miller SL, Wolfe RR. The danger of weight loss in the elderly. J Nutr Health Aging. 2008;12(7):487-91.
8. Dominguez Castro P, Reynolds CM, Kennelly S, Clyne B, Bury G, Hanlon D, et al. General practitioners' views on malnutrition management and oral nutritional supplementation prescription in the community: A qualitative study. Clin Nutr ESPEN. 2020;36:116-27.
9. Lee C, Park S. Examining Cumulative Inequality in the Association Between Childhood Socioeconomic Status and Body Mass Index From Midlife to Old Age. J Gerontol B Psychol Sci Soc Sci. 2020;75(6):1264-74.
10. Steptoe A, Zaninotto P. Lower socioeconomic status and the acceleration of aging: An outcome-wide analysis. Proc Natl Acad Sci U S A. 2020;117(26):14911-7.
11. Braun V, Clarke V. Using thematic analysis in psychology. Qualitative Research in Psychology. 2006;3(2):77-101.
12. GOV.UK. English indices of deprivation 2019 [online] available at: https://www.gov.uk/government/statistics/english-indices-of-deprivation-2019: National Statistics; 2019 [
13. Nisbett N, Harris J, Backholer K, Baker P, Jernigan VBB, Friel S. Holding no-one back: The Nutrition Equity Framework in theory and practice. Global Food Security. 2022;32:100605.
14. Verlinde E, De Laender N, De Maesschalck S, Deveugele M, Willems S. The social gradient in doctor-patient communication. International Journal for Equity in Health. 2012;11(1):12.
15. Pescud M, Pettigrew S. 'I know it's wrong, but . . .': a qualitative investigation of low-income parents' feelings of guilt about their child-feeding practices. Maternal & Child Nutrition. 2014;10(3):422-35.
16. Allen S, Rogers SN, Brown S, Harris RV. What are the underlying reasons behind socioeconomic differences in doctor-patient communication in head and neck oncology review clinics? Health Expectations. 2021;24(1):140-51.
17. Ramsay SE, Whincup PH, Morris RW, Lennon LT, Wannamethee S. Are childhood socio-economic circumstances related to coronary heart disease risk? Findings from a population-based study of older men. International Journal of Epidemiology. 2007;36(3):560-6.

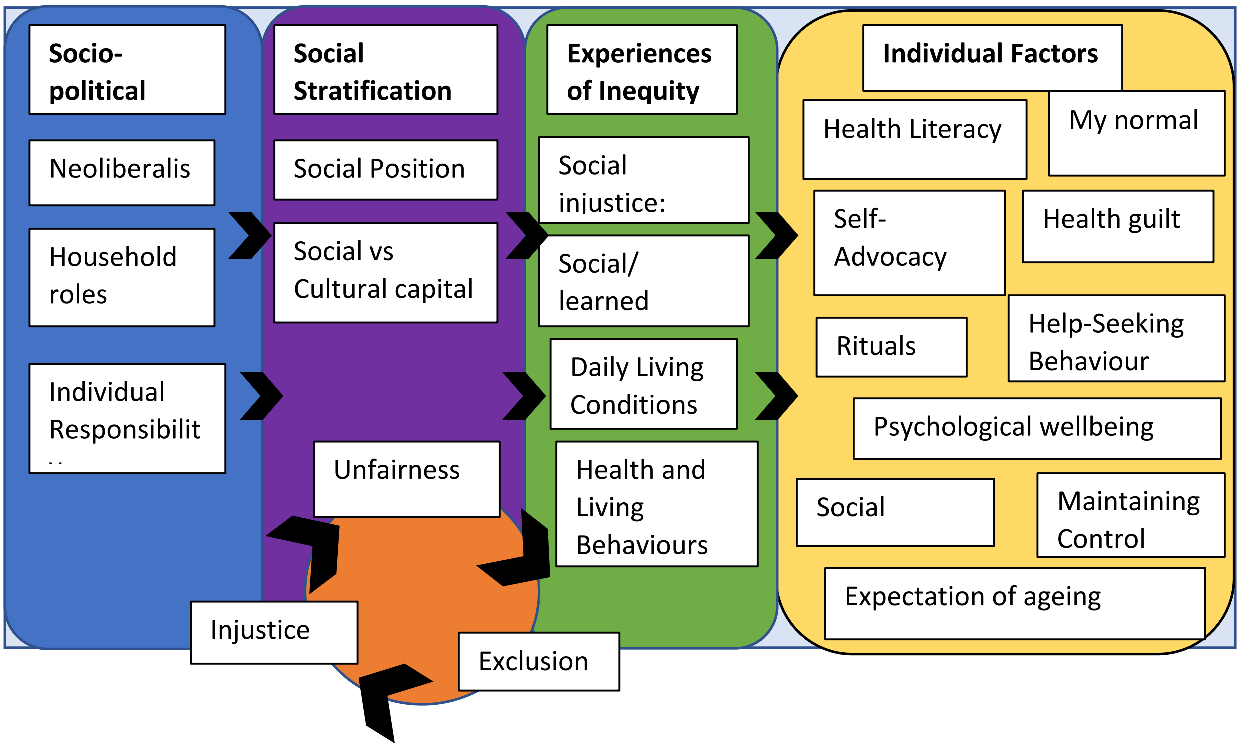

Figure 1. Summary of findings through the lens of the Nutrition Equity Framework.

Click here to access/download
Supporting Information
supplementary material.docx
