# Supplementary material for: Socioeconomic status and older adult’s experiences of weight loss: a qualitative secondary analysis
Source: PLoS One. 2025 Apr 22;20(4):e0321313. doi: 10.1371/journal.pone.0321313 (PMC12013945; doi:10.1371/journal.pone.0321313)
Supplement: Attachment — Submitted filename: Response to Reviewers 17.06 .docx [file pone.0321313.s006.docx]

Reviewer comments on manuscript:

After careful consideration, we feel that it has merit but does not fully meet PLOS ONE’s publication criteria as it currently stands. Therefore, we invite you to submit a revised version of the manuscript that addresses the points raised during the review process.

Authors response:

We thank the reviewer for their comments, and are grateful for the time they have spent reviewing

our paper. We believe that their comments have improved the quality of our manuscript, and we look forward to addressing any further feedback. Please find a point by point response to the comments in the table below, thank you.

| Please ensure that your manuscript meets PLOS ONE's style requirements, including those for file naming. | The manuscript has been formatted as per requirements |
| --- | --- |
| Please include how "at risk for frailty" was operationally defined | Extra information included in to the methods section- “ They were eligible if they were identified as having an electronic Frailty Index score of 0.36 or above” |
| Include more information on the demographic characteristics of the participants (e.g., gender, race/ethnicity) to better contextualise the findings. | Addition to the methods of more demographic data:  Study 1: Average age was 77.4 years, 14% were male. All participants were white British.  Study 2: Median age of participants was 75 years, 75% male. All participants were white British. |
| Please include information on how participants were recruited and the purpose of the primary study for which they were originally recruited | Additional information added to the methods on the sampling approach for original interview participants:  . Study 1: Participants were recruited from community-dwelling older adults referred to an integrated care centre run by the community geriatric team. They were eligible if they were identified as having an electronic Frailty Index score of 0.36 or above. An information sheet describing the study was included in the information pack provided by the service prior to attending the centre. The clinical team identified those who were interested, who were then approached by a designated member of the research team on arrival for their centre appointment, using convenience sampling.  Study 2: Participants were recruited from an inpatient tertiary cancer centre in the Yorkshire and Humber North-East of England. Participants were eligible if they were aged > 70 years with breast, colorectal, lung, prostate, head and neck, or upper gastrointestinal cancers and could give informed consent. Eligible patients were identified and approached by a member of the clinical team, and those who were interested then seen by the researcher. Convenience sampling was used to recruit participants. |
| Please carefully proofread the manuscript for spelling and grammar | The manuscript has been carefully reread as per requirements |
| Please include captions for your Supporting Information files at the end of your manuscript, and update any in-text citations to match accordingly. Please see our Supporting Information guidelines for more information: http://journals.plos.org/plosone/s/supporting-information. | Captions have been added for supporting information files |
| The statement for the aim should be revised as it contains a repetition that in my view is unnecessary. | Statement updated and revised to be clearer and more concise:  The aim of this study is to explore the impact of SES on older adult’s view of their unintentional weight loss |
| Include more recent data on  the public health expenditure  on malnutrition | Updated with more recent data:  “ In the United Kingdom, England, malnutrition is a recognised public health problem, costing 15% of the health and social care budget with older adults accounting for 52% of these total costs. (8)”  Reference: 8. Stratton R. Managing Malnutrition to improve lives and save money 2018. |
| The authors have provided a very limited review of the literature with although the argument for the social and scientific value is logical. This can be improved on by correcting minor grammatical errors and ensuring the quality of literature referenced is more recent data  As mentioned earlier, the authors should present more recent data, and consider including more studies in the presentation of the scientific and social value for this study. The comparison with other research can be improved upon. | Literature review expanded and more up to date references included with more references included in the discussion :  4. Alharbi TA, Paudel S, Gasevic D, Ryan J, Freak-Poli R, Owen AJ. The association of weight change and all-cause mortality in older adults: a systematic review and meta-analysis. Age and Ageing. 2020;50(3):697-704.  5. Sullivan DH, Bopp MM, Roberson PK. Protein-energy undernutrition and life-threatening complications among the hospitalized elderly. J Gen Intern Med. 2002;17(12):923-32.  6. Neumann SA, Miller MD, Daniels L, Crotty M. Nutritional status and clinical outcomes of older patients in rehabilitation. J Hum Nutr Diet. 2005;18(2):129-36.  7. Hussain SM, Newman AB, Beilin LJ, Tonkin AM, Woods RL, Neumann JT, et al. Associations of Change in Body Size With All-Cause and Cause-Specific Mortality Among Healthy Older Adults. JAMA Network Open. 2023;6(4):e237482.  8. Stratton R. Managing Malnutrition to improve lives and save money 2018.  13. Bullock AF, Patterson MJ, Paton LW, Currow DC, Johnson MJ. Malnutrition, sarcopenia and cachexia: exploring prevalence, overlap, and perceptions in older adults with cancer. European Journal of Clinical Nutrition. 2024.  15. Kheifets M, Goshen A, Goldbourt U, Witberg G, Eisen A, Kornowski R, et al. Association of socioeconomic status measures with physical activity and subsequent frailty in older adults. BMC Geriatrics. 2022;22(1):439.  21. Jackson SE, Holter L, Beeken RJ. ‘Just because I’m old it doesn’t mean I have to be fat’: a qualitative study exploring older adults’ views and experiences of weight management. BMJ Open. 2019;9(2):e025680.  22. Simons AMW, Houkes I, Koster A, Groffen DAI, Bosma H. The silent burden of stigmatisation: a qualitative study among Dutch people with a low socioeconomic position. BMC Public Health. 2018;18(1).  30. Turner AJ, Francetic I, Watkinson R, Gillibrand S, Sutton M. Socioeconomic inequality in access to timely and appropriate care in emergency departments. Journal of Health Economics. 2022;85:102668.  31. Arpey NC, Gaglioti AH, Rosenbaum ME. How Socioeconomic Status Affects Patient Perceptions of Health Care: A Qualitative Study. Journal of Primary Care & Community Health. 2017;8(3):169-75.  32. Coupe N, Cotterill S, Peters S. Tailoring lifestyle interventions to low socio-economic populations: a qualitative study. BMC Public Health. 2018;18(1):967.  33. Eggink E, Hafdi M, Hoevenaar-Blom MP, Richard E, Charante EPMv. Attitudes and views on healthy lifestyle interventions for the prevention of dementia and cardiovascular disease among older people with low socioeconomic status: a qualitative study in the Netherlands. BMJ Open. 2022;12(2):e055984.  34. Sargent-Cox K. Ageism: we are our own worst enemy. International Psychogeriatrics. 2017;29(1):1-8.  35. Fernández-Jiménez C, Dumitrache CG, Rubio L, Ruiz-Montero PJ. Self-perceptions of ageing and perceived health status: the mediating role of cognitive functioning and physical activity. Ageing and Society. 2024;44(3):622-41.  36. Velaithan V, Tan M-M, Yu T-F, Liem A, Teh P-L, Su TT. The Association of Self-Perception of Aging and Quality of Life in Older Adults: A Systematic Review. The Gerontologist. 2023;64(4).  37. McCoy CA, Johnston E, Hogan C. The impact of socioeconomic status on health practices via health lifestyles: Results of qualitative interviews with Americans from diverse socioeconomic backgrounds. Social Science & Medicine. 2024;344:116618.  38. Siu J, Giskes K, Turrell G. Socio-economic differences in weight-control behaviours and barriers to weight control. Public Health Nutrition. 2011;14(10):1768-78. |
| Please confirm that this does not alter your adherence to all PLOS ONE policies on sharing data and materials, by including the following statement: ""This does not alter our adherence to PLOS ONE policies on sharing data and materials.” (as detailed online in our guide for authors http://journals.plos.org/plosone/s/competing-interests). If there are restrictions on sharing of data and/or materials, please state these. Please note that we cannot proceed with consideration of your article until this information has been declared.  Please include your updated Competing Interests statement in your cover letter; we will change the online submission form on your behalf. | This has been included in the cover letter and updated on the manuscript to reflect that this does not alter adherence |
| This could be improved with comparison with more studies, although few studies have looked directly at the subject, there is a lot of research in behaviour change and diet in weight gain. | Thank you, we have added a paragraph in discussion with more recent references and brief discussion of the literature on weight gain  Our findings are consistent with previous research identifying the messages from adulthood, including concerns about weight gain, are carried forward into old age [9, 10, 21]. Older adults from lower socioeconomic backgrounds were more likely to feel stigma with regards to their diet and assign self-blame for choices that deviate from public health messages. Despite the good intentions of health policy, this can lead to feelings of shame and inferiority, particularly in those from lower socioeconomic decile [22-26] Previous research into weight gain has demonstrated that in older adults, the prevalence of frail obesity was higher in those from lower SES and increased in prevalence by 1.49 for each additional social disadvantage.[27] Having a higher social status has been shown to buffer the psychological impact of weight stigma. [28] [24] Carrying this forward into older age, those from lower socioeconomic deciles carried stigma and guilt into their later life, mitigating concern they had for unintentional weight loss. |
